# Leukocyte telomere length and depression, anxiety and stress and adjustment disorders in primary health care patients

**DOI:** 10.1186/s12888-017-1308-0

**Published:** 2017-04-24

**Authors:** Xiao Wang, Kristina Sundquist, Anna Hedelius, Karolina Palmér, Ashfaque A. Memon, Jan Sundquist

**Affiliations:** Center for Primary Health Care Research, Lund University/Region Skåne, Malmö, Sweden

**Keywords:** Anxiety, Depression, Stress and adjustment disorders, Telomere length

## Abstract

**Background:**

The primary aim was to examine possible differences in telomere length between primary health care patients, with depression, anxiety or stress and adjustment disorders, and healthy controls. The second aim was to examine the association between telomere length and baseline characteristics in the patients. The third aim was to examine the potential effects of the 8-week treatments (mindfulness-based group therapy or treatment as usual, i.e. mostly cognitive-based therapy) on telomere length, and to examine whether there was a difference in the potential effect on telomere length between the two groups.

**Methods:**

A total of 501 individuals including 181 patients (aged 20–64 years), with depression, anxiety and stress and adjustment disorders, and 320 healthy controls (aged 19–70 years) were recruited in the study. Patient data were collected from a randomized controlled trial comparing mindfulness-based group therapy with treatment as usual. We isolated genomic DNA from blood samples, collected at baseline and after the 8-week follow-up. Telomere length was measured by quantitative real-time (qRT)-PCR.

**Results:**

Telomere length was significantly shorter in the patients (mean = 0.77 ± 0.12,), compared to the controls (mean = 0.81 ± 0.14) (*p* = 0.006). The difference in telomere length remained significant after controlling for age and sex. Old age, male sex and being overweight were associated with shorter telomere length. There was no significant difference in telomere length between baseline and at the 8-week follow-up in any of the treatment groups and no difference between the two groups.

**Conclusion:**

Our findings confirm that telomere length, as compared with healthy controls, is shortened in patients with depression, anxiety and stress and adjustment disorders. In both groups (mindfulness-based group therapy or treatment as usual), the telomere length remained unchanged after the 8-week treatment/follow-up and there was no difference between the two groups.

**Trial registration:**

(ClinicalTrials.gov ID: NCT01476371) Registered November 11, 2011.

**Electronic supplementary material:**

The online version of this article (doi:10.1186/s12888-017-1308-0) contains supplementary material, which is available to authorized users.

## Background

Depression, anxiety, and stress and adjustment disorders are common psychiatric disorders, with an estimated prevalence that varies between 12 and 32% of the total medical practice consultations in primary care in Europe [1, 2]. These illnesses place a large economic burden on society and cause emotional and social difficulties for patients and their relatives and can often result in problems such as a loss of income and a poorer quality of life. In addition, individuals with depression, anxiety or stress and adjustment disorders also have an increased risk of developing age-related illnesses, such as cardiovascular disease, cancer, diabetes and Parkinson’s disease [3–10]. Previous studies have shown that lifestyle, environmental factors, and biological processes are involved in the associations between psychiatric disorders and age-related illnesses [11–14]. However, the mechanisms underlying these relationships are still not fully understood. In recent years, the association between psychiatric disorders and accelerated biological aging, evaluated by the emerging biomarker (telomere length), has gained much attention [4].

Telomeres are DNA-protein complexes that cap the chromosomal DNA ends, which preserve genomic integrity and stability [15, 16]. Telomeres shorten during somatic cell division because DNA polymerase is unable to fully replicate the 3′ end of DNA. This process can be reversed by an enzyme (telomerase) that is only active in certain replicating tissues, such as male germ cells and activated lymphocytes, stem cells and cancer cells [17, 18]. In normal human cells, telomerase levels are insufficient to maintain telomere length during cell division. When telomeres reach a critically short length, cell growth becomes limited and undergoes cellular senescence or apoptosis [4, 19]. Oxidative stress and chronic inflammation accelerate telomere attribution, resulting in replicative senescence and organ degeneration [11, 20, 21]. Telomere length reflects the cumulative damage from those exposure factors and can be used as a potential indicator of biological aging. Shorter telomere length has been linked to the development of a variety of age-related diseases, such as cancer [22, 23], cardiovascular disease [24, 25] and diabetes [26]; diseases that are associated with common psychiatric disorders [3–10].

Previous studies have found that telomere length is significantly shorter in patients with depression or mood disorders as compared to controls [4, 12, 13, 27–31]. The associations between telomere length and severity of depression have also been investigated, but the results are inconsistent among these studies [11–13, 32]. This may be because most previous studies have only included major/severe depression, in contrast to the mild and moderate symptoms in patients with depression and anxiety diagnosed in primary health care. To our knowledge, only one study, which was done by Verhoeven et al., has included primary health care patients. However, that study only included patients with major depression [12]. To explore the association between telomere length and mild and moderate depression and anxiety will help us to understand the underlying mechanisms of these disorders, which may lead to better prevention strategies in order to avoid age-related illnesses. Moreover, most studies have only measured telomere length at a single point in time. Very few studies have examined the potential changes of telomere length after treatment for mental disorders [13, 33]. Hartmann et al. found that major depression is associated with shortened telomeres but independent from therapy with antidepressants and/or ECT [13]. Martinsson et al. reported that long-term lithium treatment in patients with bipolar disorder is associated with longer leukocyte telomere length [33]. Epel et al. proposed that some forms of meditation, for example, mindfulness meditation training, could have beneficial effects on telomere length by reducing cognitive stress and increasing positive states of mind [34]. In addition, cognitive behavioral therapy (CBT) has been recognized as an effective way of treating depressive disorders [35] and CBT might also have beneficial effects on telomere length [36]. A recent study reported that mindfulness-based and supportive therapeutic interventions in breast cancer survivors helped maintain telomere length over the 3-month intervention period in contrast to usual care [37]. Previous studies, including a meta-analytic review, have shown that mindfulness therapies and meditation are associated with increased telomerase activity [38–40]. Data from a recently published study of ours showed that mindfulness group therapy and treatment as usual (TAU, mostly individual-based CBT) reduced psychiatric symptoms in patients with depression, anxiety, and stress and adjustment disorders, and that there was no significant difference in treatment response between the two groups [41]. However, the potential treatment effect on molecular biomarkers related to chronic diseases and biological aging, such as telomere length, with a specific focus on the potential difference in molecular biomarkers after different types of psychological treatment has not been fully investigated. We, therefore, aimed to examine whether mindfulness therapy and TAU could have a positive influence on telomere length in patients with relatively mild/moderate forms of depression, anxiety and stress and adjustment disorders treated in primary health care and whether there is a difference in the potential treatment effect on telomere length between the two therapies. This has not been examined previously and the potential clinical relevance of our study is high. If mindfulness training or TAU has an effect on biological aging, it may improve the physical health among patients with common psychiatric disorders treated in primary health care in addition to the improvement in psychiatric symptoms [41].

The present study builds on a previously published randomized controlled trial (RCT), by our group, where we included patients with depression, anxiety or stress and adjustment disorders from 16 primary health care centers in order to compare the effects of an 8-week mindfulness-based group therapy with TAU(mostly CBT) on psychiatric symptoms [41]. In the present analysis, we used data on telomere length collected in the RCT at baseline and after the 8-week follow-up. The first aim was to examine possible differences in telomere length between patients with depression, anxiety or stress and adjustment disorders and healthy controls. The second aim was to examine the association between telomere length and baseline characteristics in the patients with depression, anxiety or stress and adjustment disorders. The third aim was to examine the potential effects of the 8-week treatments (mindfulness-based group therapy or TAU) on telomere length and to examine whether there was a difference in the potential effect on telomere length between the two groups.

## Methods

### Study population

The study population included 181 patients (age 20–64 years) that had been diagnosed with depression, anxiety or stress and adjustment disorders. All the patients were recruited from 16 primary health care centers in an RCT comparing mindfulness group therapy with TAU. A more detailed description of the study design, patient information and sample collection has been provided in previous publications [41, 42]. Patients were recruited between Jan 4, 2012 and March 22, 2012 in urban and rural areas in the county of Scania (In Swedish: Skåne) in southern Sweden. Newly diagnosed patients and those who had a history of psychiatric disorders were both eligible. However, only those who sought treatment for their psychiatric disorder during the recruitment period were considered for inclusion in the study.

Patients who fulfilled the inclusion criteria were included in the study. All clinical diagnoses were made by doctors at the 16 primary health care centers: 1. One or more of the following ICD-10 psychiatric diagnoses: F32.0, mild depressive episode; F32.1, moderate depressive episode; F32.9, depressive episode, unspecified; F33.0, recurrent depressive disorder, current episode mild; F33.1, recurrent depressive disorder, current episode moderate; F41.0, panic disorder; F41.1, generalized anxiety disorder; F41.2, mixed anxiety and depressive disorder; F41.3, other mixed anxiety disorders; F41.8, other specified anxiety disorders; F41.9, anxiety disorder, unspecified; F43.2, adjustment disorders; F43.8, other reactions to severe stress; and F43.9, reaction to severe stress, unspecified; 2. Age 20–64 years; 3. Ability to speak and read Swedish; and 4. A score of ≥10 on the Patient Health Questionnaire (PHQ)-9 or ≥7 on the Hospital Anxiety and Depression Scale (HADS) or a total score on the Montgomery-Åsberg Depression Rating Scale (MADRS-S) between 13 and 34 (mild to moderate depression). Patients who met any of the following criteria were excluded from the study: severe personality disorder, risk of suicide, pregnancy, thyroid disease, current psychotherapy of any kind and participation in any other psychiatric intervention study. Each patient completed three self-rated questionnaires (above-mentioned PHQ-9, HADS-A/HADS-D, and MADRS-S) at baseline and after 8 weeks of follow-up. The patients received antidepressants and tranquilizers if deemed necessary. Blood samples were collected at the same time as the assessment of self-rated scales, before and after 8-week follow-up. The control group, which was comprised of 320 volunteer blood donors (age 19–70 years), was recruited in 2015 from the blood center in Malmö. All the individuals met the criteria in Sweden for blood donation. In order to donate blood in Sweden, one needs to be able to speak, read and understand Swedish. One also needs to have a Swedish personal identity number, an approved ID document and to be a healthy person between the ages of 18–60. Exclusions can be made for healthy individuals older than 60 years. Before donation, the individual must answer several health-related questions [43]. For example, donation is not allowed among individuals with severe asthma and/or receiving treatment with steroids tablets. Mild or moderate depression is not an exclusion criterion. Blood donors in Sweden are not paid for donating blood.

### Leukocyte telomere length (LTL) measurement

Mean telomere length was determined from peripheral blood leukocytes. Genomic DNA was extracted from 200 μL frozen blood using a commercially available kit; QiAamp96 DNA Blood Kit (Qiagen, Hilden, Germany). The quantities of DNA samples were determined by Nanodrop (ND-2000, Thermo Scientific, USA) and then normalized to 5 ng/ul in TE buffer (0.1 mM EDTA, 10 mM Tris [pH 7.5]) containing *Escherichia coli* DNA (~3 ng/μl; carrier DNA without telomeres). The DNA was heated at 95 °C for 30 min in a thermal cycler, directly followed by 1 min on ice, spun down briefly at 1000×g, 4 °C, and then saved at 4 °C until the PCR was performed (<3 days).

LTL was measured by real-time PCR based on the method reported by Cawthon with modification [44–46], as this is the only method available for large-scale measurement [47, 48]. The relative telomere to single copy gene (T/S) ratio reflects the average length of the telomeres. A single copy reference gene, β-hemoglobin (HBG), was used to normalize the quantity of the input DNA. PCR reactions were performed separately in telomere and HBG 384-well plates. The plates were identical for DNA amount, reaction volume and sample order. All samples and reference DNA were run in triplicate using 20 ng DNA. A negative control was included in each run.

For each plate, the master mix was prepared with the following reagents: 1xPCR buffer, 0.2 mM dNTP (Fermentas), 0.2X SYBR Green I (Roche Diagnostics, Germany) and 1.25 Units AmpliTaq Gold DNA polymerase (Applied Biosystems). In addition, the master mix for telomere plate contained 1.75 mM MgCL_2_, 2.5 mM DTT and telomere primers as follows: Tel 1: 5′-GGTTTTTGAGGGTGAGGGTGAGGGTGAGGGTGAGGGT-3′, 100 nM; Tel 2: 5′- TCCCGACTATCCCTATCCCTTCCCTATCCCTATCCCTA-3′, 900 nM. The HBG PCR mix contained 2 mM MgCL_2_, 5 mM DTT and the following primers: HBG3: 5′-TGTGCTGGCCCATCACTTTG-3′, 400 nM; HBG4: 5′-ACCAGCCACCACTTTCTGATAGG-3′, 400 nM. PCR was performed on CFX 384 Real-Time PCR Detection System (Bio-Rad, Hercules, CA, USA), with the thermal cycling profile for telomere amplification: 95 °C 10min + 25 cycle*(95 °C 15s + 56 °C 1min) + melting curve. For HBG, 95 °C 10min + 32 cycle*(95 °C 15s + 58 °C 30s + 72 °C 30s) + melting curve.

LTL was quantified by qPCR using the standard curve method [26, 49]. A standard curve using the reference DNA (from Jurkat cell line) diluted serially by 2-fold per dilution to produce seven concentrations of 0.3 to 20 ng/ul. Those seven reference DNA samples were included in each assay plate. T and S concentration were calculated according to the standard curve. Standard curves were generated using the Bio-Rad CFX Manager software v. 2.0. R2 for each standard curve was >0.99 for both the telomere and HBG reaction. The corresponding PCR efficiencies were 91.2% for telomere length and 92.3% for HBG. Standard deviations from the triplicate were accepted at <0.4. The inter-coefficients of variance (CV) for T/S ratio was less than 5%.

### Statistical analysis

For all the patients and healthy controls, we present the mean and SD for age and telomere length, median and interquartile range (IQR) for MADRS-S, HAD-D, HAD-A and PHQ-9. Sex, BMI, smoking status, alcohol status, education and antidepressant and/or tranquilizer use are presented as numbers and percentages (Table 1). Between-group comparisons of the age and telomere length were done by independent sample t-test and of sex by Chi-square test (Table 1).

**Table 1:** Characteristics of the study population at baseline (*n* = 501)

Variables	Patients (*n* = 181)	Control subjects (*n* = 320)	*P*-value
Age, years			
Mean (SD)	41.9 (11.1)	44.6 (12.5)	0.02^a^
Sex, n (%)			
Male	22 (12)	197 (62)	<0.0001^b^
Female	159 (88)	123 (38)	
Telomere length			
Mean (SD)	0.77 (0.12)	0.81 (0.14)	0.006^a^
BMI, n (%)^c^			
Underweight	2 (1.1)		
Normal weight	70 (39)	N/A	N/A
Overweight	56 (31)		
Obese	44 (24)		
Smoking status, n (%)^d^			
Yes	27 (15)	N/A	N/A
No	151 (83)		
Alcohol status, n (%)^e^			
Standard size drinks per week			
< 1	100 (55)		
1–4	54 (30)	N/A	N/A
> =5	22 (12)		
Education, n (%)^f^			
Low	15 (8)		
Middle	72 (40)	N/A	N/A
High	92 (51)		
Antidepressants, n (%)^g^			
Yes	63 (35)	N/A	N/A
No	102 (56)		
Tranquilizers, n (%)^h^			
Yes	27 (15)	N/A	N/A
No	131 (72)		
Baseline MADRS-S			
Median score (IQR)	20 (14–25)	N/A	N/A
Baseline HAD-D			
Median score (IQR)	8 (6–11)	N/A	N/A
Baseline HAD-A			
Median score (IQR)	12 (9–15)	N/A	N/A
Baseline PHQ-9			
Median score (IQR)	13 (9–17)	N/A	N/A

*Abbreviations*: *SD* Standard Deviation, *BMI* Body Mass Index, *IQR* Interquartile Range, *N/A* Not Available

Education: Low = 0–9 years; Middle = 10–12 years; High = More than 12 years

^a^ *P*-value is given for the comparison of patients and control subjects using independent sample t-test

^b^ *P*-value is based on Chi-square test

^c^9 (5%) had missing on BMI

^d^3 (2%) had missing on smoking status

^e^5 (3%) had missing on alcohol status

^f^2 (1%) had missing on education

^g^16 (9%) had missing on antidepressants

^h^23 (13%) had missing on tranquilizers

Univariate and multivariate (adjusted for age and sex) linear regression were used to test the differences between patients and controls at baseline. The association between telomere length and age and sex was examined in univariate models and adjusted for the other variables (Table 2). The associations between telomere length and baseline characteristics of patients were analyzed by univariate and multivariate linear regression (Table 3).

**Table 2:** Differences in telomere length between patients (*n* = 181) and controls (*n* = 320) at baseline

	Univariate			Multivariate		
Variables	β	*P*-value^a^	95% CI	β	*P*-value^a^	95% CI
Patients vs controls	−0.04	0.006	−0.06; −0.01	−0.07	<0.001^b^	−0.10; −0.05
Age	−0.005	<0.001	−0.006; −0.004	−0.005	<0.001^c^	−0.006;-0.004
Female vs male	0.03	0.01	0.007; 0.06	0.05	<0.001^d^	0.03; 0.08

^a^Difference tested by a linear regression model

^b^Adjusted for age and sex

^c^Adjusted for group and sex

^d^Adjusted for group and age

**Table 3:** Associations between telomere length and characteristics of patients at baseline (*n* = 181)

	Univariate			Adjusted for age and sex		
Variables	β	*P*-value^a^	95% CI	β	*P*-value^a^	95% CI
Age	−0.04	<0.001	−0.006; −0.003			
Female vs male	0.06	0.04	0.003; 0.11			
BMI						
Underweight	0.13	0.15	−0.04; 0.30	0.08	0.33	−0.08; 0.24
Normal weight	Ref			Ref		
Overweight	−0.05	0.01	−0.10; −0.01	−0.04	0.04	−0.08; −0.001
Obese	−0.03	0.20	−0.08; 0.02	−0.02	0.30	−0.07; 0.02
Smoking status (yes vs no)	−0.002	0.94	−0.05; 0.05	−0.007	0.78	−0.05; 0.04
Alcohol status						
Standard size drinks per week						
< 1	Ref			Ref		
1–4	0.02	0.37	−0.02; 0.06	0.02	0.36	−0.02; 0.06
> =5	0.04	0.15	−0.01; 0.10	0.05	0.08	−0.006; 0.10
Education						
Low	−0.07	0.04	−0.14; −0.002	−0.03	0.41	−0.09; 0.04
Middle	−0.04	0.04	−0.08; −0.002	−0.03	0.15	−0.06; 0.009
High	Ref			Ref		
Pharmacotherapy^b^ (yes vs no)	−0.005	0.79	−0.04; 0.03	−0.007	0.68	−0.04; 0.03
MADRS-S	0.002	0.11	−0.0005; 0.004	0.002	0.19	−0.00007; 0.004
HAD-D	0.002	0.45	−0.003; 0.007	0.003	0.16	−0.001; 0.008
HAD-A	0.004	0.12	−0.001; 0.009	0.001	0.66	−0.004; 0.006
PHQ-9	0.003	0.05	−0.00005; 0.006	0.002	0.13	−0.0006; 0.005

^a^Association tested by a linear regression model

^b^Antidepressants and/or tranquilizers

**Fig. 1:**
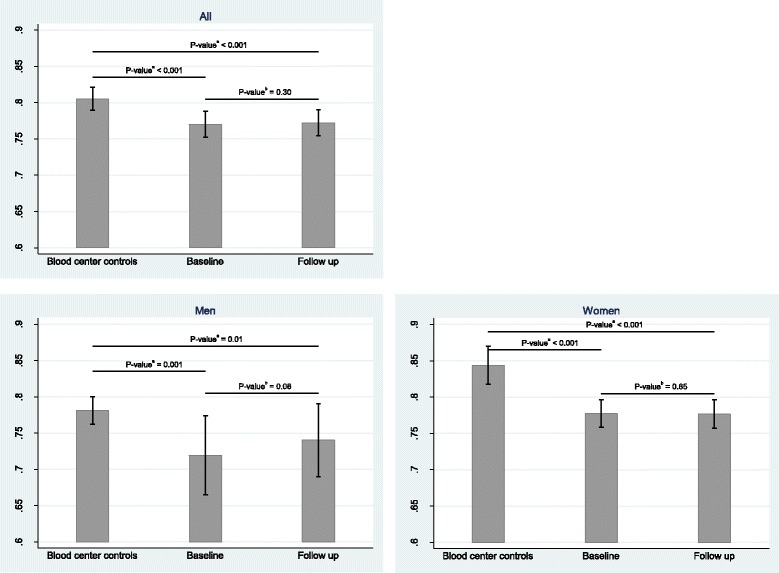
Mean values and 95% confidence intervals of telomere length for patients and control subjects (at baseline and follow-up) together with *p*-values (*n* = 501). All analyses are adjusted for age. ^a^Difference tested by independent sample t-test. ^b^Difference tested by paired t-test

Within group comparisons between baseline and follow-up was done by paired t-test (Table 4), stratified by mindfulness treatment and TAU. Due to the correlation of measurements within individuals, we used a random-intercept linear regression model (univariate and adjusted for age, sex, BMI and pharmacological treatment) to examine the difference in effect between mindfulness and TAU (Table 5). In Fig. 1, we show the mean telomere length with 95% confidence intervals for control subjects and, for patients, at both baseline and follow-up. Independent sample t-test was used to examine the difference between patients and controls at baseline. Paired t-test was used to test the difference between baseline and follow-up for patients. All analyses were adjusted for age and the graphs were shown for the whole population and stratified by sex. Within group comparisons between baseline and follow-up were examined separately for men and women and for patients with and without pharmacological treatment (Additional file 1: Table S1). The correlation between telomere length and age was analyzed with the Pearson correlation (ρ) and shown in a scatterplot together with a fitted line from a linear regression (Additional file 2: Figure S1). STATA version 14 (StataCorp LP) was used for all statistical analyses.

**Table 4:** Telomere length for patients at baseline and after follow up in the mindfulness and treatment as usual groups (*n* = 181)

	Baseline		Follow up		Difference	
Variables	Mean (SD)	n	Mean (SD)	n	Mean (SD)	*P*-value^a^
All	0.77 (0.12)	177	0.77 (0.12)	177	−0.005 (0.06)	0.30
Mindfulness	0.76 (0.11)	88	0.77 (0.12)	88	−0.007 (0.05)	0.21
Treatment as usual	0.78 (0.14)	89	0.78 (0.13)	89	−0.002 (0.06)	0.79

^a^Difference tested by paired t-test

**Table 5:** Analysing treatment effect between mindfulness and treatment as usual (*n* = 181)

	Univariate			Multivariate^b^		
Variables	β	*P*-value^c^	95% CI	β	*P*-value^c^	95% CI
Mindfulness vs treatment as usual	−0.02	0.33	−0.06; 0.01	−0.02	0.34	−0.05; 0.02
Follow up vs baseline (time)	0.001	0.83	−0.01; 0.01	0.002	0.78	−0.01; 0.01
Treatment group*time^a^	0.006	0.50	−0.01; 0.02	0.006	0.48	−0.01; 0.02

^a^Interaction effect (treatment effect) between treatment group and time

^b^Adjusted for age, sex, BMI and antidepressants and/or tranquilizers

^c^Treatment effect tested by a random-intercept linear regression model using GLLAMM

## Results

Population characteristics and differences in telomere length between patients and controls at baseline.

The characteristics of the study population are shown in Table 1. The mean age in the patient group (*n* = 181) was 41.9 years (SD = 11.1) and in the control group 44.6 years (SD = 12.5). The patient group included a lower proportion of males than the control group. In the patient group, most participants (92%) had more than 10 years of education and were non-smokers (83%) or non-alcohol abusers. The median scores for MADRS-S, HADS-A/HADS-D and PHQ-9 at baseline indicated mild to moderate symptoms of depression and/or anxiety. After treatment, the median scores decreased, indicating none to mild symptoms (data not shown in tables) [41].

The telomere length was normally distributed and significantly inversely correlated with age (Additional file 2: Figure S1) (ρ = −0.44, *p* < 0.0001). Females had a longer telomere length than males (β = 0.05, *p* < 0.001) (Table 2). Telomere length (mean ± SD) was significantly shorter in the patients than in the controls at baseline (0.77 ± 0.12 vs. 0.81 ± 0.14; *p* = 0.006) (Table 1). We further used linear regression to test the difference in telomere length between patients and controls at baseline; the difference in telomere length between the two groups remained significant after controlling for age and sex (ß = −0.07, *p* < 0.001) (Table 2).

### Association between telomere length and baseline characteristics in patients

Next, we applied linear regression analysis to investigate the association between telomere length and baseline characteristics in patients. Table 3 shows that age and sex are associated with telomere length (*p* < 0.001 and *p* = 0.04, respectively). Overweight patients had significantly shorter telomere length than those with normal weight, after adjusting for age and sex (β = −0.04, *p* = 0.04). However, this association was not observed in the obese group (β-0.02, *p* = 0.30). No significant association was observed between telomere length and the rest of the baseline characteristics. Interestingly, there was no significant association between telomere length and the severity of the symptoms measured by any of the scales (Table 3).

### The potential effect of the 8-week treatment on telomere length

Paired T-tests were used to examine the potential effect of the treatment on telomere length in the patients. This analysis showed that the telomere length remained unchanged after the 8-week treatment in both the mindfulness and the TAU group (*p* = 0.21 and *p* = 0.79, respectively, Table 4) and there was no difference between the two groups (*p* = 0.34, Table 5). This was examined with a random-intercept linear regression model, univariate and adjusted for age, sex, BMI and pharmacotherapy (Table 4 and Table 5), the latter representing antidepressants and/or tranquilizers use. When stratifying for sex, men had slightly longer telomere lengths after the 8-week follow-up. However, this increase was not significant (*p* = 0.08), perhaps due to insufficient sample size in men. In women, no change in telomere length was observed after treatment (*p* = 0.65) (Additional file: Table S1 and Fig. 1). No significant changes in telomere length were found when stratifying for pharmacotherapy (Additional file 1: Table S1).

## Discussion

The main findings of the present study were that telomere length was significantly shorter in the patients compared to the controls (aim 1). Old age, male sex and being overweight were associated with shortened telomere length (aim 2). The telomere length remained unchanged after the 8-week treatment in both the mindfulness and the TAU group and there was no difference between the two groups (aim 3), which represents a novel contribution.

Our results regarding shortened telomere length, in the patients compared with the healthy controls, are in line with previous findings [13, 29, 30, 50]. The biological mechanisms underlying the shortened telomere length in patients with these psychiatric disorders remain to be elucidated. Although the pathophysiology of depression, anxiety and stress and adjustment disorders has multiple facets, chronic life stress may have a role in the etiology of some cases of depression and anxiety. Previous studies have also shown that psychological stress is significantly related with higher oxidative stress, lower telomerase activity and shortened telomere length [14, 51, 52]. Telomeres are nucleoprotein structures, consisting of stretches of repetitive DNA with a high number of G-C repetitions that are highly sensitive to oxidative stress [53]. Oxidative stress is associated with accelerated telomere shortening in vitro [54]. Therefore, a possible explanation behind shortened telomere length in patients with depression, anxiety and stress and adjustment disorders is that stressful life events may increase oxidative stress and thus decrease telomere length.

It should, however, be noted that no significant association was found between telomere length and the severity of disease evaluated by self-rated scales. This finding is in line with Hartmann et al. who found that the telomere length of patients with major depression was shortened but not related to the severity of disease [13].

Although we found that old age, male sex and being overweight were associated with shortened telomere length in the patient group, we did not find significant associations between telomere length and the other variables (smoking, alcohol drinking, educational level and treatment with antidepressants and/or tranquilizers) in the analyses of the associations between telomere length and baseline characteristic within the patient group.

The most important and novel finding of the present study is that the telomere length remained unchanged after the 8-week treatment in both the mindfulness and the TAU group and that there was no difference between the two groups. Our study is the first to evaluate the possible effects of both mindfulness-based group therapy and TAU (mostly individual CBT) on telomere length in patients with mild to moderate depression, anxiety and stress and adjustment disorders. We hypothesized that mindfulness training or TAU may have a beneficial effect on telomere length in our study population of patients with depression, anxiety and stress and adjustment disorders. Although more studies are needed, our findings may indicate that telomere length is maintained with mindfulness-based group therapy as well as with individual CBT. In line with our results, Carlson et al. reported that mindfulness-based and supportive therapeutic interventions helps maintain telomere length over a 3-month intervention period in contrast to usual care, where a decreased telomere length was observed [37]. However, the relatively short follow-up (8-weeks) may not be a long enough duration to observe significant changes in telomere length over time. It is possible that a longer follow-up and continued mindfulness training is needed to detect robust effects on telomere length. Hoge et al. found that individuals with more than 4 years of practice in Loving-Kindness Meditation had longer telomeres [55].

Our study has several strengths. We obtained the results from an RCT including sampling of biological markers in 181 patients with mild to moderate depression as well as patients with anxiety or stress and adjustment disorders. These patients are highly representative of the most common psychiatric disorders in the general population, i.e. those who are treated in primary health care. This represents a novel contribution to existing research as previous studies have mainly included patients with severe depression. However, our study also has some limitations that should be considered when interpreting our results. First, we do not have information on potential psychiatric disorders in the control group. However, blood donors are normally healthier than the general population and we do not expect that even a possible inclusion of a relatively low percentage of potential cases with common psychiatric disorders would have affected the conclusions of this study. Previous research has suggested that social factors, ethnicity and lifestyle factors, such as smoking, BMI, and physical inactivity, may affect telomere length [56–58]. Only the variables age and sex were available in the control population; a possible limitation in some of the analyses. In order to check whether our patient group was similar to the general population in the county of Scania (i.e. the geographic region where our study was conducted) we compared the prevalence of certain lifestyle factors among our patients with the prevalence of these factors in a public health report conducted in Scania in 2012 [59]. The mean BMI among the participants (mean age 46.5 y) in the public health report was 25.6, which is relatively close to the mean BMI among our patients, i.e. 26.4. The corresponding percentages for daily smoking were 12.4% in the public health report and 15.0% in our patient group.

## Conclusions

The present study shows that patients with depression, anxiety or stress and adjustment disorders have shorter telomere length than healthy controls; a finding that is in line with previous research. However, the most important finding is that the telomere length remained unchanged after the 8-week treatment in both the mindfulness and the TAU group and that there was no difference between the two groups. These findings lend insight to the design of future studies, which should follow participants over a longer time span to detect robust effects on telomere length in patients with depression, anxiety or stress and adjustment disorders.

## Additional files

Additional file 1: Table S1. Telomere length for patients at baseline and follow-up after mindfulness and treatment as usual, stratified for sex and pharmacotherapy. (DOC 66 kb)

Additional file 2: Figure S1. Pearson’s correlation and *p*-value for association between telomere length and age in patients and control subjects at baseline. (DOC 41 kb)

## References

[1] Vazquez-Barquero JL, Garcia J, Simon JA, Iglesias C, Montejo J, Herran A, Dunn G (1997). Mental health in primary care. An epidemiological study of morbidity and use of health resources. Br J Psychiatry.

[2] Nordstrom A, Bodlund O (2008). Every third patient in primary care suffers from depression, anxiety or alcohol problems. Nord J Psychiatry.

[3] Irwin MR, Miller AH (2007). Depressive disorders and immunity: 20 years of progress and discovery. Brain Behav Immun.

[4] Lindqvist D, Epel ES, Mellon SH, Penninx BW, Revesz D, Verhoeven JE, Reus VI, Lin J, Mahan L, Hough CM (2015). Psychiatric disorders and leukocyte telomere length: underlying mechanisms linking mental illness with cellular aging. Neurosci Biobehav Rev.

[5] Nemeroff CB, Musselman DL, Evans DL (1998). Depression and cardiac disease. Depress Anxiety.

[6] Evans DL, Charney DS, Lewis L, Golden RN, Gorman JM, Krishnan KR, Nemeroff CB, Bremner JD, Carney RM, Coyne JC (2005). Mood disorders in the medically ill: scientific review and recommendations. Biol Psychiatry.

[7] Kyrou I, Kollia N, Panagiotakos D, Georgousopoulou E, Chrysohoou C, Tsigos C, Randeva HS, Yannakoulia M, Stefanadis C, Papageorgiou C, et al. Association of depression and anxiety status with 10-year cardiovascular disease incidence among apparently healthy Greek adults: the ATTICA study. Eur J Prev Cardiol. 2017;24(2):145–52. 10.1177/2047487316670918 27671771

[8] Svensson E, Farkas DK, Gradus JL, Lash TL, Sorensen HT (2016). Adjustment disorder and risk of Parkinson's disease. Eur J Neurol.

[9] Roy S, Chakraborty A, Ghosh C, Banerjee B (2015). Systematic analysis of integrated Gene functional network of four chronic stress-related lifestyle disorders. Genome Integrity.

[10] Gradus JL, Farkas DK, Svensson E, Ehrenstein V, Lash TL, Milstein A, Adler N, Sorensen HT (2015). Associations between stress disorders and cardiovascular disease events in the Danish population. BMJ Open.

[11] Wolkowitz OM, Mellon SH, Epel ES, Lin J, Dhabhar FS, Su Y, Reus VI, Rosser R, Burke HM, Kupferman E (2011). Leukocyte telomere length in major depression: correlations with chronicity, inflammation and oxidative stress--preliminary findings. PLoS One.

[12] Verhoeven JE, Revesz D, Epel ES, Lin J, Wolkowitz OM, Penninx BW (2014). Major depressive disorder and accelerated cellular aging: results from a large psychiatric cohort study. Mol Psychiatry.

[13] Hartmann N, Boehner M, Groenen F, Kalb R (2010). Telomere length of patients with major depression is shortened but independent from therapy and severity of the disease. Depress Anxiety.

[14] Epel ES, Blackburn EH, Lin J, Dhabhar FS, Adler NE, Morrow JD, Cawthon RM (2004). Accelerated telomere shortening in response to life stress. Proc Natl Acad Sci U S A.

[15] Blackburn EH (2001). Switching and signaling at the telomere. Cell.

[16] de Lange T (2005). Shelterin: the protein complex that shapes and safeguards human telomeres. Genes Dev.

[17] Blackburn EH (2000). Telomere states and cell fates. Nature.

[18] Beyne-Rauzy O, Prade-Houdellier N, Demur C, Recher C, Ayel J, Laurent G, Mansat-De Mas V (2005). Tumor necrosis factor-alpha inhibits hTERT gene expression in human myeloid normal and leukemic cells. Blood.

[19] Shay JW, Wright WE (2007). Hallmarks of telomeres in ageing research. J Pathol.

[20] De Meyer T, Rietzschel ER, De Buyzere ML, Van Criekinge W, Bekaert S (2008). Studying telomeres in a longitudinal population based study. Front Biosci.

[21] von Zglinicki T (2002). Oxidative stress shortens telomeres. Trends Biochem Sci.

[22] Wu X, Amos CI, Zhu Y, Zhao H, Grossman BH, Shay JW, Luo S, Hong WK, Spitz MR (2003). Telomere dysfunction: a potential cancer predisposition factor. J Natl Cancer Inst.

[23] Willeit P, Willeit J, Mayr A, Weger S, Oberhollenzer F, Brandstatter A, Kronenberg F, Kiechl S (2010). Telomere length and risk of incident cancer and cancer mortality. JAMA.

[24] Calado RT, Young NS (2009). Telomere diseases. N Engl J Med.

[25] Brouilette SW, Moore JS, McMahon AD, Thompson JR, Ford I, Shepherd J, Packard CJ, Samani NJ (2007). Telomere length, risk of coronary heart disease, and statin treatment in the west of Scotland primary prevention study: a nested case-control study. Lancet.

[26] Zhao J, Zhu Y, Lin J, Matsuguchi T, Blackburn E, Zhang Y, Cole SA, Best LG, Lee ET, Howard BV (2014). Short leukocyte telomere length predicts risk of diabetes in american indians: the strong heart family study. Diabetes.

[27] Garcia-Rizo C, Fernandez-Egea E, Miller BJ, Oliveira C, Justicia A, Griffith JK, Heaphy CM, Bernardo M, Kirkpatrick B (2013). Abnormal glucose tolerance, white blood cell count, and telomere length in newly diagnosed, antidepressant-naive patients with depression. Brain Behav Immun.

[28] Hoen PW, de Jonge P, Na BY, Farzaneh-Far R, Epel E, Lin J, Blackburn E, Whooley MA (2011). Depression and leukocyte telomere length in patients with coronary heart disease: data from the heart and soul study. Psychosom Med.

[29] Lung FW, Chen NC, Shu BC (2007). Genetic pathway of major depressive disorder in shortening telomeric length. Psychiatr Genet.

[30] Simon NM, Smoller JW, McNamara KL, Maser RS, Zalta AK, Pollack MH, Nierenberg AA, Fava M, Wong KK (2006). Telomere shortening and mood disorders: preliminary support for a chronic stress model of accelerated aging. Biol Psychiatry.

[31] Cai N, Chang S, Li Y, Li Q, Hu J, Liang J, Song L, Kretzschmar W, Gan X, Nicod J (2015). Molecular signatures of major depression. Curr Biol.

[32] Karabatsiakis A, Kolassa IT, Kolassa S, Rudolph KL, Dietrich DE (2014). Telomere shortening in leukocyte subpopulations in depression. BMC Psychiatry.

[33] Martinsson L, Wei Y, Xu D, Melas PA, Mathe AA, Schalling M, Lavebratt C, Backlund L (2013). Long-term lithium treatment in bipolar disorder is associated with longer leukocyte telomeres. Transl Psychiatry.

[34] Epel E, Daubenmier J, Moskowitz JT, Folkman S, Blackburn E (2009). Can meditation slow rate of cellular aging? Cognitive stress, mindfulness, and telomeres. Ann N Y Acad Sci.

[35] Oei TP, Bullbeck K, Campbell JM (2006). Cognitive change process during group cognitive behaviour therapy for depression. J Affect Disord.

[36] Basu N, Skinner HG, Litzelman K, Vanderboom R, Baichoo E, Boardman LA (2013). Telomeres and telomere dynamics: relevance to cancers of the GI tract. Expert Rev Gastroenterol Hepatol.

[37] Carlson LE, Beattie TL, Giese-Davis J, Faris P, Tamagawa R, Fick LJ, Degelman ES, Speca M (2015). Mindfulness-based cancer recovery and supportive-expressive therapy maintain telomere length relative to controls in distressed breast cancer survivors. Cancer.

[38] Schutte NS, Malouff JM (2014). A meta-analytic review of the effects of mindfulness meditation on telomerase activity. Psychoneuroendocrinology.

[39] Daubenmier J, Lin J, Blackburn E, Hecht FM, Kristeller J, Maninger N, Kuwata M, Bacchetti P, Havel PJ, Epel E (2012). Changes in stress, eating, and metabolic factors are related to changes in telomerase activity in a randomized mindfulness intervention pilot study. Psychoneuroendocrinology.

[40] Lavretsky H, Epel ES, Siddarth P, Nazarian N, Cyr NS, Khalsa DS, Lin J, Blackburn E, Irwin MR (2013). A pilot study of yogic meditation for family dementia caregivers with depressive symptoms: effects on mental health, cognition, and telomerase activity. Int J Geriatr Psychiatry.

[41] Sundquist J, Lilja A, Palmer K, Memon AA, Wang X, Johansson LM, Sundquist K (2015). Mindfulness group therapy in primary care patients with depression, anxiety and stress and adjustment disorders: randomised controlled trial. Br J Psychiatry.

[42] Wang X, Sundquist K, Hedelius A, Palmer K, Memon AA, Sundquist J (2015). Circulating microRNA-144-5p is associated with depressive disorders. Clin Epigenetics.

[43] Blood donation in Sweden https://geblodnu/english-engelska/.

[44] Cawthon RM (2002). Telomere measurement by quantitative PCR. Nucleic Acids Res.

[45] Cawthon RM, Smith KR, O'Brien E, Sivatchenko A, Kerber RA (2003). Association between telomere length in blood and mortality in people aged 60 years or older. Lancet.

[46] Nordfjall K, Osterman P, Melander O, Nilsson P, Roos G (2007). hTERT (−1327)T/C polymorphism is not associated with age-related telomere attrition in peripheral blood. Biochem Biophys Res Commun.

[47] Bojesen SE (2013). Telomeres and human health. J Intern Med.

[48] Rode L, Nordestgaard BG, Bojesen SE (2015). Peripheral blood leukocyte telomere length and mortality among 64,637 individuals from the general population. J Natl Cancer Inst.

[49] Lin J, Epel E, Cheon J, Kroenke C, Sinclair E, Bigos M, Wolkowitz O, Mellon S, Blackburn E (2010). Analyses and comparisons of telomerase activity and telomere length in human T and B cells: insights for epidemiology of telomere maintenance. J Immunol Methods.

[50] Wolkowitz OM, Epel ES, Reus VI, Mellon SH (2010). Depression gets old fast: do stress and depression accelerate cell aging?. Depress Anxiety.

[51] Gururajan A, Clarke G, Dinan TG, Cryan JF (2016). Molecular biomarkers of depression. Neurosci Biobehav Rev.

[52] Szebeni A, Szebeni K, DiPeri T, Chandley MJ, Crawford JD, Stockmeier CA, Ordway GA (2014). Shortened telomere length in white matter oligodendrocytes in major depression: potential role of oxidative stress. Int J Neuropsychopharmacol.

[53] Kawanishi S, Oikawa S (2004). Mechanism of telomere shortening by oxidative stress. Ann N Y Acad Sci.

[54] von Zglinicki T, Saretzki G, Ladhoff J, d'Adda di Fagagna F, Jackson SP (2005). Human cell senescence as a DNA damage response. Mech Ageing Dev.

[55] Hoge EA, Chen MM, Orr E, Metcalf CA, Fischer LE, Pollack MH, De Vivo I, Simon NM (2013). Loving-kindness meditation practice associated with longer telomeres in women. Brain Behav Immun.

[56] Weischer M, Bojesen SE, Nordestgaard BG (2014). Telomere shortening unrelated to smoking, body weight, physical activity, and alcohol intake: 4,576 general population individuals with repeat measurements 10 years apart. PLoS Genet.

[57] Lynch SM, Peek MK, Mitra N, Ravichandran K, Branas C, Spangler E, Zhou W, Paskett ED, Gehlert S, DeGraffinreid C (2016). Race, ethnicity, psychosocial factors, and telomere length in a multicenter setting. PLoS One.

[58] Revesz D, Verhoeven JE, Milaneschi Y, Penninx BW (2016). Depressive and anxiety disorders and short leukocyte telomere length: mediating effects of metabolic stress and lifestyle factors. Psychol Med.

[59] Folkhälsorapport FHS. http://www.socialstyrelsen.se/, 2012.

