# Supplementary material for: Leukocyte telomere length and depression, anxiety and stress and adjustment disorders in primary health care patients
Source: BMC Psychiatry. 2017 Apr 24;17:148. doi: 10.1186/s12888-017-1308-0 (PMC5404668; doi:10.1186/s12888-017-1308-0)
Supplement: Supplementary file 1 — Telomere length for patients at baseline and follow-up after mindfulness and treatment as usual, stratified for sex and pharmacotherapy. (DOC 66 kb) [file 12888_2017_1308_MOESM1_ESM.doc]

**Additional file 1**

| **Table S1.** Telomere length for patients at baseline and follow-up after mindfulness and treatment as usual, stratified for sex and pharmacotherapy (n = 181) | | | | | | |
| --- | --- | --- | --- | --- | --- | --- |
|  |  | |  | |  | |
|  | **Baseline** | | **Follow-up** | | **Difference** | |
|  | **Mean (SD)** | **n** | **Mean (SD)** | **n** | **Mean (SD)** | **P-value^a^** |
| **Men** |  |  |  |  |  |  |
| All | 0.72 (0.12) | 22 | 0.74 (0.11) | 22 | -0.02 (0.05) | 0.08 |
| Mindfulness | 0.72 (0.11) | 14 | 0.75 (0.10) | 14 | -0.03 (0.06) | 0.07 |
| Treatment as usual | 0.71 (0.16) | 8 | 0.72 (0.13) | 8 | -0.005 (0.05) | 0.79 |
|  |  |  |  |  |  |  |
| **Women** |  |  |  |  |  |  |
| All | 0.77 (0.12) | 155 | 0.78 (0.12) | 155 | -0.002 (0.06) | 0.65 |
| Mindfulness | 0.77 (0.11) | 74 | 0.77 (0.11) | 74 | -0.002 (0.05) | 0.64 |
| Treatment as usual | 0.78 (0.13) | 81 | 0.78 (0.13) | 81 | -0.001 (0.06) | 0.83 |
|  |  |  |  |  |  |  |
| **Patients with pharmacotherapy^b^** |  |  |  |  |  |  |
| All | 0.77 (0.12) | 74 | 0.78 (0.12) | 74 | -0.01 (0.06) | 0.06 |
| Mindfulness | 0.76 (0.11) | 32 | 0.77 (0.12) | 32 | -0.01 (0.05) | 0.14 |
| Treatment as usual | 0.78 (0.13) | 42 | 0.79 (0.12) | 42 | -0.01 (0.06) | 0.23 |
|  |  |  |  |  |  |  |
| **Patients without pharmacotherapy^b^** |  |  |  |  |  |  |
| All | 0.77 (0.12) | 92 | 0.77 (0.12) | 92 | 0.002 (0.05) | 0.73 |
| Mindfulness | 0.76 (0.11) | 49 | 0.77 (0.11) | 49 | -0.004 (0.04) | 0.56 |
| Treatment as usual | 0.78 (0.14) | 43 | 0.77 (0.14) | 43 | 0.008 (0.06) | 0.37 |

^a^Difference tested by paired t-test.

^b^Antidepressants and/or tranquilizers.
