# Supplementary material for: Leukocyte telomere length and depression, anxiety and stress and adjustment disorders in primary health care patients
Source: BMC Psychiatry. 2017 Apr 24;17:148. doi: 10.1186/s12888-017-1308-0 (PMC5404668; doi:10.1186/s12888-017-1308-0)
Supplement: Supplementary file 2 — Pearson’s correlation and p-value for association between telomere length and age in patients and control subjects at baseline. (DOC 41 kb) [file 12888_2017_1308_MOESM2_ESM.doc]

**Additional file 2**

**Figure S1.** Pearson’s correlation and p-value for association between telomere length and age in patients and control subjects at baseline (n = 501)

ρ = Pearson’s correlation.

^a^Predicted line of telomere length by age estimated by a linear regression model.
